# Postoperative Transient Neurogenic Claudication After Lumbar Endoscopic Decompression: The Role of Floseal in Minimally Invasive Spine Surgery

**DOI:** 10.7759/cureus.73083

**Published:** 2024-11-05

**Authors:** Helena Krogman, Bryan Stevens, Meena Kanhai, Matthew Meroney, Sanjeev Kumar, Amir Jafari

**Affiliations:** 1 Department of Anesthesiology, College of Medicine, University of Florida, Gainesville, USA; 2 Department of Pain Management, College of Medicine, University of Florida, Gainesville, USA; 3 Department of Anesthesiology/Pain Medicine, College of Medicine, University of Florida, Gainesville, USA

**Keywords:** chronic pain management, lumbar endoscopic decompression, minimally-invasive spine surgery, surgical case report, unusual postoperative course

## Abstract

The shift toward minimally invasive procedures in American healthcare has transformed spine surgery, particularly with the rise of endoscopic techniques. Endoscopic spine surgery enables the treatment of conditions like lumbar spinal stenosis, offering advantages such as reduced tissue trauma, smaller incisions, and quicker recovery compared to traditional open surgery. Spinal stenosis, where the spinal canal narrows and compresses nerves, often results in significant pain, numbness, or weakness in the lower extremities. Despite the benefits of endoscopic decompression, it brings unique challenges, particularly regarding intraoperative bleeding control. Hemostatic agents, such as Floseal (Baxter, Deerfield, IL), a gelatin matrix, are commonly used to manage bleeding during surgery. However, their use in endoscopic procedures within confined spaces introduces considerations not seen in open surgeries. This case report presents a patient with lumbar stenosis who underwent endoscopic decompression and developed postoperative neurogenic claudication, illustrating both the outcome and the use of Floseal in minimally invasive spine surgeries.

## Introduction

Minimally invasive techniques have revolutionized modern surgical practices, offering alternatives to traditional open procedures with reduced recovery times and fewer complications. In the field of spine surgery, these advancements have made complex interventions, such as spinal decompression, possible through endoscopic approaches. Endoscopic spine surgery, in particular, is increasingly preferred for the treatment of conditions like lumbar spinal stenosis, where the narrowing of the spinal canal leads to nerve compression, causing symptoms such as pain, numbness, and weakness in the lower back and legs.

Lumbar stenosis, one of the most common causes of radicular pain in older adults, has traditionally been treated through open surgical techniques. However, endoscopic decompression has gained traction due to its ability to minimize tissue damage, reduce postoperative recovery time, and decrease surgical risks. Despite these advantages, endoscopic techniques bring new complexities, including challenges related to intraoperative bleeding management and postoperative outcomes that are not as well-studied as those for open surgery.

Floseal (Baxter, Deerfield, IL), a gelatin-thrombin matrix hemostatic agent, is commonly employed in endoscopic procedures to control intraoperative bleeding. Its gelatin component absorbs water and swells to fit the bleeding site, while thrombin enhances clot formation. This hemostatic process is critical in surgical procedures, where bleeding must be controlled delicately without causing additional tissue damage. However, the use of Floseal in confined spaces, such as the spine, requires careful consideration of its volume and degradation timeline, as improper management may lead to complications.

This case report examines a patient who underwent endoscopic lumbar decompression and experienced postoperative neurogenic claudication, highlighting the use of Floseal. The report underscores the importance of understanding and tailoring the use of hemostatic agents in minimally invasive spine surgeries and adds to the evolving body of knowledge in this field.

## Case presentation

A male in his early seventies presented with chronic lumbar radicular pain radiating to both lower extremities, accompanied by numbness in the left leg and intermittent bilateral lower extremity weakness. The patient's symptoms were most pronounced in the L4-5 dermatomal distribution, with greater severity on the left side. Although he reported subjective weakness, no objective findings of muscle weakness were observed during the initial physical examination.

**Figure 1:**
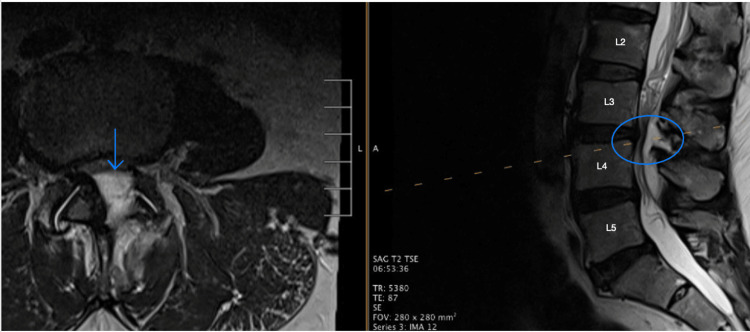
Preoperative MRI indicating the central canal and lateral recess stenosis at L3-4. Shown in blue is the original pathology: central canal and lateral recess stenosis at L3-4, which is responsible for the patient's symptoms. MRI, magnetic resonance imaging

A stepwise conservative treatment plan was followed, beginning with physical therapy, non-steroidal anti-inflammatory drugs (NSAIDs), and acetaminophen. When these measures failed, the patient underwent multiple epidural steroid injections. Lumbar magnetic resonance imaging (MRI) revealed significant findings, including ligamentum flavum thickening at L3-4, resulting in moderate to severe spinal canal narrowing, as well as mild narrowing at L4-5 (Figure 1). Given the severity of his symptoms and the lack of improvement with conservative treatments, the patient opted for endoscopic nerve decompression.

Although conservative treatment is often preferred for mild to moderate stenosis, the severity of the patient’s symptoms, particularly the bilateral recess effacement at L3-4, made surgical intervention more appropriate. Due to the bilateral nature of the stenosis, an interlaminar approach was chosen over a transforaminal one. Intraoperatively, minor bleeding occurred, but hemostasis was achieved using Trigger Flex cautery and 10 mm of Floseal. Postoperatively, the patient’s neurological examination was consistent with his preoperative baseline, and he was discharged the same day. However, seven days after the procedure, the patient continued to report symptoms of neurogenic claudication, requiring assistance with ambulation, the patient reported symptoms of neurogenic claudication, requiring assistance with ambulation. Despite this, he denied any signs of cauda equina syndrome or infection and demonstrated full strength on physical examination. Due to the persistence of his pain after the operation, a transforaminal epidural steroid injection (TFESI) was administered to alleviate his symptoms. It is relatively common for patients to experience a recurrence of radiculopathy following endoscopic surgery, often due to postoperative inflammation. This was believed to be the cause of the patient's symptoms, leading to the decision to perform bilateral TFESIs at the L3-4 and L4-5 levels. The injections were given a few days before a repeat MRI was ordered after they failed to relieve the patient’s symptoms.

**Figure 2:**
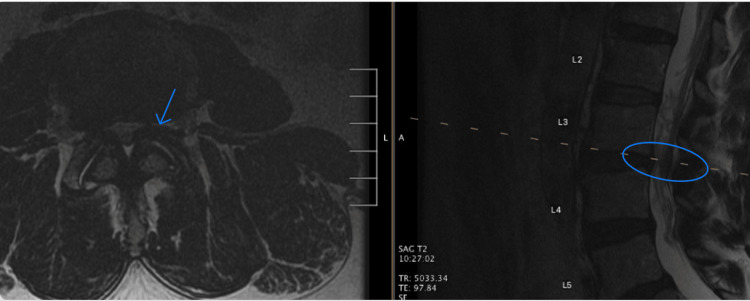
MRI showing Floseal aggregate in the epidural space at the decompressed level - one week postoperative. Shown in blue is the Floseal aggregate, which was originally misdiagnosed by the neuroradiologist as an epidural abscess. MRI, magnetic resonance imaging

Unfortunately, the injections failed to provide relief, and a repeat MRI lumbar spine was ordered, as shown in Figure 2. Initially, the reading radiologist noted a 6.1-cm epidural fluid collection, suspecting a possible hematoma or possible abscess. After discussion with the neuroradiologist, the radiology report was amended to include a slowly reabsorbing collection of thrombin aggregate used intraoperatively.

While Floseal is commonly used in open procedures, its use in endoscopic surgery within confined spaces can lead to temporary compressive symptoms due to the volume of injectate. The patient gradually improved, and approximately one month after surgery, he was able to walk unaided. By eight weeks postoperatively, he reported being completely pain-free, with full recovery sustained at the time of writing.

## Discussion

Endoscopic spine surgery was developed as a safer alternative to conventional open spine surgery for the treatment of degenerative spinal diseases. While it has gained popularity as a minimally invasive method, this case highlights that endoscopic spine surgery is still an evolving field with unique challenges. Some well-known complications include dural tears and intraoperative bleeding, which require effective hemostasis [1]. Floseal, a gelatin-based hemostatic matrix, is commonly used to control bleeding in such procedures [2,3].

An important consideration with the use of Floseal in endoscopic spine surgery is that minimally invasive procedures, such as endoscopic surgery, present challenges not encountered in traditional open approaches. In open surgeries, the volume of injectate is less of a concern, as surrounding bony structures are often removed, creating more space. However, in endoscopic spine surgeries, where these structures remain intact, careful consideration of the injectate’s physiological half-life and volume is paramount [4].

Floseal, a biocompatible hemostatic agent, is absorbed and broken down by the body over time. Its gelatin matrix component is typically resorbed within weeks, as denatured collagen is hydrolyzed by the body's proteolytic enzymes, such as matrix metalloproteinases. These enzymes break down the gelatin into amino acids and peptides, which are either reused by the body or excreted. The thrombin component, a protein, also degrades through enzymatic processes, losing its activity shortly after initiating clot formation. The breakdown and absorption of Floseal are highly variable, influenced by factors such as the patient’s physiology and the nature of the surgery. According to the product information provided by Baxter (Deerfield, IL), the manufacturer of Floseal, the gelatin matrix is typically reabsorbed within six to eight weeks after application as was the case for the patient described in this case report. It’s also important to note that Floseal can expand by 10%-20% upon contact with bodily fluids, potentially exacerbating compressive symptoms. While there is no definitive guideline on the safest volume for use in endoscopic decompression, ensuring adequate hemostasis without causing compression is vital.

In this patient, the use of excess Floseal created a postoperative complication that initially masked the success of the endoscopic decompression. The patient experienced persistent pain, and postoperative MRI revealed a fluid collection that was initially misinterpreted as an epidural hematoma or abscess [5]. Fortunately, after consulting with a neuroradiologist, it was identified as a slowly resorbing thrombin aggregate from the Floseal application. Neurosurgical interventions were avoided, and the patient’s symptoms gradually improved once Floseal was fully absorbed, allowing the decompression to take effect.

## Conclusions

This case highlights a previously undocumented postoperative consideration in endoscopic spine surgery. As this minimally invasive approach becomes more widespread, it is crucial to report and understand adverse events to improve patient outcomes. This case underscores the importance of recognizing that certain benign conditions, such as a Floseal matrix, can mimic more serious pathologies like epidural hematoma or abscess, potentially leading to unnecessary interventions. Given the limited clinical guidelines for the use of hemostatic agents in endoscopic spinal procedures, these findings suggest the need for a more tailored approach to achieving hemostasis in confined surgical spaces.

The lack of clear clinical guidelines for the use of hemostatic agents in endoscopic spinal procedures highlights the need for careful consideration of both volume and resorption rates, particularly in confined spaces. Clear communication within the team, including neuroradiologists, about the use of Floseal and its appearance in postoperative imaging is essential to prevent misdiagnosis.

Although limited to a single patient, this case underscores the importance of ongoing documentation of unique postoperative outcomes in endoscopic spine surgery. These experiences are essential for enhancing patient safety and optimizing surgical outcomes as minimally invasive techniques evolve.
